# Growth in eligibility criteria content and failure to accrue among National Cancer Institute (NCI)‐affiliated clinical trials

**DOI:** 10.1002/cam4.5276

**Published:** 2022-11-18

**Authors:** John S. Peterson, Deborah Plana, Danielle S. Bitterman, Skyler Bryce Johnson, Hugo J. W. L. Aerts, Benjamin Harris Kann

**Affiliations:** ^1^ Department of Radiation Oncology Huntsman Cancer Institute Salt Lake City Utah USA; ^2^ Harvard Medical School Boston Massachusetts USA; ^3^ Department of Radiation Oncology Dana‐Farber Cancer Institute/Brigham and Women's Hospital, Harvard Medical School Boston Massachusetts USA; ^4^ Artificial Intelligence in Medicine (AIM) Program Brigham and Women's Hospital, Harvard Medical School Boston Massachusetts USA; ^5^ Radiology and Nuclear Medicine Maastricht University Maastricht The Netherlands

## Abstract

**Background:**

Cancer trial accrual is a national priority, yet up to 20% of trials fail to accrue. Trial eligibility criteria growth may be associated with accrual failure. We sought to quantify eligibility criteria growth within National Cancer Institute (NCI)‐affiliated trials and determine impact on accrual.

**Methods:**

Utilizing the Aggregated Analysis of ClinicalTrials.gov, we analyzed phase II/III interventional NCI‐affiliated trials initiated between 2008 and 2018. Eligibility criteria growth was assessed via number of unique content words within combined inclusion and exclusion criteria. Association between unique word count and accrual failure was evaluated with multivariable logistic regression, adjusting for known predictors of failure. Medical terms associated with accrual failure were identified via natural language processing and categorized.

**Results:**

Of 1197 trials, 231 (19.3%) failed due to low accrual. Accrual failure rate increased with eligibility criteria growth, from 11.8% in the lowest decile (12–112 words) to 29.4% in the highest decile (445–750 words). Median eligibility criteria increased over time, from 214 (IQR [23, 282]) unique content words in 2008 to 417 (IQR [289, 514]) in 2018 (*r* ^2^ = 0.73, *P* < 0.001). Eligibility criteria growth was independently associated with accrual failure (OR: 1.09 per decile, 95% CI [1.03–1.15], *p* = 0.004). Eighteen exclusion criteria categories were significantly associated with accrual failure, including renal, pulmonary, and diabetic, among others (Bonferroni‐corrected *p* < 0.001).

**Conclusions:**

Eligibility criteria content growth is increasing dramatically among NCI‐affiliated trials and is strongly associated with accrual failure. These findings support national initiatives to simplify eligibility criteria and suggest that further efforts are warranted to improve cancer trial accrual.

## 1 INTRODUCTION

Cancer clinical trial enrollment is a national priority.^1^ Significant resources are spent on encouraging trial accrual, recognizing that clinical trial enrollment may lead to improved survival over standard of care.^1^ Despite these efforts, it is estimated that only 3% of adults with cancer in the United States participate in clinical trials and 24% of trials fail to reach more than half of their recruitment goals.^2^, ^3^, ^4^

Reasons for failure to accrue are complex, and might include changes in eligibility criteria characteristics.^2^, ^5^ Restrictive eligibility criteria have been implicated in low accrual, and in widening demographic disparities between the patients enrolled in clinical trials and the larger population, which the results of the studies are meant to serve.^6^, ^7^ Overall, it is estimated that one in five cancer patients is ineligible to participate in clinical trials based on eligibility criteria.^2^

A prior study investigating a cooperative group's lung cancer trial protocols showed that eligibility criteria were increasing.^8^, ^9^ Broader trends in eligibility criteria among cancer trials remain unexplored, and the relationship between trial accrual failure and eligibility criteria growth remains undefined. Recent developments in natural language processing (NLP), a branch of artificial intelligence that extracts quantitative data from free text, have proven effective in analyzing large volume medical text in situations that would otherwise require prohibitive and error‐prone manual curation processes.^10^ Leveraging publicly available clinical trial data and NLP tools, we sought to investigate the impact of eligibility criteria content changes on cancer trial accrual and identify criteria characteristics that are associated with accrual failure.

## 2 METHODS

### 2.1 Data source and selection

A retrospective study of clinical trial data gathered from the Aggregated Analysis of ClinicalTrials.gov (AACT) and the Cancer Trials Support Unit (CTSU) websites was conducted. The AACT is a daily‐updated, public online relational database containing all protocols and results data on every clinical trial listed on Clinicaltrials.Gov.^11^ The Clinical Trial Support Unit (CTSU) hosts up‐to‐date information on cancer clinical trials supported by the National Cancer Institute (NCI).^12^ Given the public nature of all data used, no institutional review board approval was required. The AACT and CTSU data were extracted in their entirety on February 2, 2021 for analysis, yielding 365,685 unique studies. AACT data were curated and organized by individual study identifier, and 20 descriptive variables were extracted, including the entire text of each trial's eligibility criteria. The 1197 trials included for analysis were initiated between January 1, 2008 and December 31, 2018, were interventional Phase II or III, affiliated with the National Cancer Institute (NCI), and were first classified as either “Completed” or “Failed” as per the study definition. “Failed trials” were defined as those whose current status was “Terminated,” “Suspended,” “Withdrawn,” or those who failed to achieve 50% of target enrollment after 2 years (Appendix 1). Accrual failure, the primary endpoint of the study, was defined as failed trials whose reason for failure was specified as an accrual problem and/or trials that failed to achieve 50% of target enrollment after 2 years. This enrollment cut‐off was based on previous literature demonstrating that most clinical trials fail if they do not reach half of the target enrollment after 2 years.^3^, ^4^ Trials were excluded if they were not yet completed and also did not meet our definition of failure.

### 2.2 Analysis of trial accrual failures

The cause of trial failure was determined using the “why_stopped” data field, which provides a brief explanation of why a study was halted or terminated (Supplementary Tables S1). Previous research has identified specific trial characteristics to be associated with NCI‐affiliated trial failure.^4^ We adapted the search terms used in this work to identify cancer types and grouped trials that studied common solid and liquid tumors (Supplementary Table S2); common solid tumors were defined as breast, colorectal, lung, and prostate and common liquid cancers were leukemia, lymphoma, and myeloma.^4^ To identify all clinical trials that used an NCI‐approved targeted therapy, we collected the names of all NCI‐approved cancer drugs from www.cancer.gov/about‐cancer/treatment/drugs (accessed January 13, 2021) and then cross‐checked them within each trial's intervention Medical Subject Headings (MeSH). We identified trials that required tissue samples by searching for the phrases “biopsy,” “sample,” “specimen,” “paraffin‐embedded,” “paraffin embedded,” and “tumor tissue” in the eligibility criteria. Trials done in a metastatic setting were found by searching for “metasta” and “stage iv” in the trial title or trial description.

### 2.3 Analysis of eligibility criteria content

Eligibility criteria content was quantified using number of unique content words, utilizing the natural language processing (NLP) package, Natural Language Tool Kit (NLTK) with Python version 3.9.2 (Python Software Foundation, Python Language Reference, available at http://www.python.org).^13^ Specifically, we tokenized individual, unique words in the eligibility criteria content of each trial and removed “stop words,” or common words with little interpretive value (e.g., “and,” “the,” “at”).^14^ The number of unique content words in each trial's eligibility criteria was then counted.

The median number of unique content words in eligibility criteria was plotted for clinical trials initiated in each year. The fraction of clinical trials that failed due to low accrual was similarly plotted by initiation year. Accrual failure rate was analyzed by decile of eligibility criteria unique word count. The association between eligibility criteria content and accrual failure rate was analyzed using univariable and multivariable logistic regression. Multivariable regression included the following variables that had been previously associated with accrual failure among NCI‐affiliated clinical trials between 2000 and 2013: tumor site, biopsy requirement, use of approved targeted therapy, radiation therapy, clinical trial phase, and metastatic setting.^4^ Odds ratios with 95% confidence intervals were calculated, and a *p* value of <0.05 was considered statistically significant. An analogous analysis was also performed using the number of individual eligibility criteria as a surrogate for complexity, instead of unique word count. Additionally, we conducted regression analyses investigating the association between accrual failure and unique content words within inclusion criteria and exclusion criteria separately. Statistical analyses were performed using R software, version 3.6.0 (R Project, Vienna, Austria).

A sensitivity analysis was performed using a more permissive definition of “completion”, including all trials whose status was “Completed,” or “Recruiting,” or “Active, not recruiting” with greater than 50% accrual, so as to test whether the trends identified would remain significant (Appendix 2).

### 2.4 Medical term association with accrual failure and feature importance

The specialized biomedical NLP library, ScispaCy was used to identify biomedical terms within the exclusion criteria in each clinical trial that could be associated with accrual failure (Appendix 1). Upon qualitative review of trial exclusion and inclusion criteria individually, it was observed that there was substantial variation in the way in which the inclusion criteria were written—sometimes inclusively, sometimes exclusively, and sometimes neutral. This variation was far less prevalent among exclusion criteria, which were primarily list of barriers to enrollment. For this reason, we chose to conduct this analysis only on exclusion criteria to capture terms that were restrictive to enrollment. ScispaCy is an open‐source software package built on the Python‐based spaCy model and retrained on large volumes of biomedical text from the United Medical Language System (UMLS), RxNorm, GeneOntology, Medical Subject Headings (MeSH), and the Human Phenotype Ontology.^15^ Several specialized pipelines are available through ScispaCy; we opted to utilize the model “en_core_sci_md,” which offers name entity recognition for approximately 360,000 terms and 50,000 word vectors. The en_core_sci_md pipeline identified 12,870 unique n‐grams of five words or less. To increase processing speed, all n‐grams that only appeared in the dataset once were excluded leaving 5909 n‐grams. Clinical terms that appeared significantly more frequently in accrual failure trials were identified via two‐sided chi‐squared tests of independence with post hoc Bonferroni correction for multiple hypothesis testing at a significance level of *p* = 6.7 × 10^−6^. The resulting biomedical terms were then reviewed by authors (JP, BK) for medical relevancy and qualitatively grouped into thematic clinical categories (Table 2).

Given the hypothesized complex and non‐linear relationships between exclusion clinical categories and accrual failure, we chose to model accrual failure and analyze category predictive importance with machine learning, using a gradient‐boosted trees classifier via the Python XGboost package.^16^ As model input, we utilized the clinical categories referenced above as categorical variables and also included a continuous variable representing the sum of the total number of medical terms in a given trial. The data were randomly divided into a training set (80%) and test set (20%), stratified by accrual failure. Model hyperparameters were optimized using a grid search with 10‐fold cross validation (Appendix 1). The model with the highest performing area under the receiver operating characteristic curve (AUC) was chosen for testing. We calculated each feature's importance via the Gini (impurity‐based) importance.^17^

## 3 RESULTS

### 3.1 Trial failure rate and reasons

**FIGURE 1:**
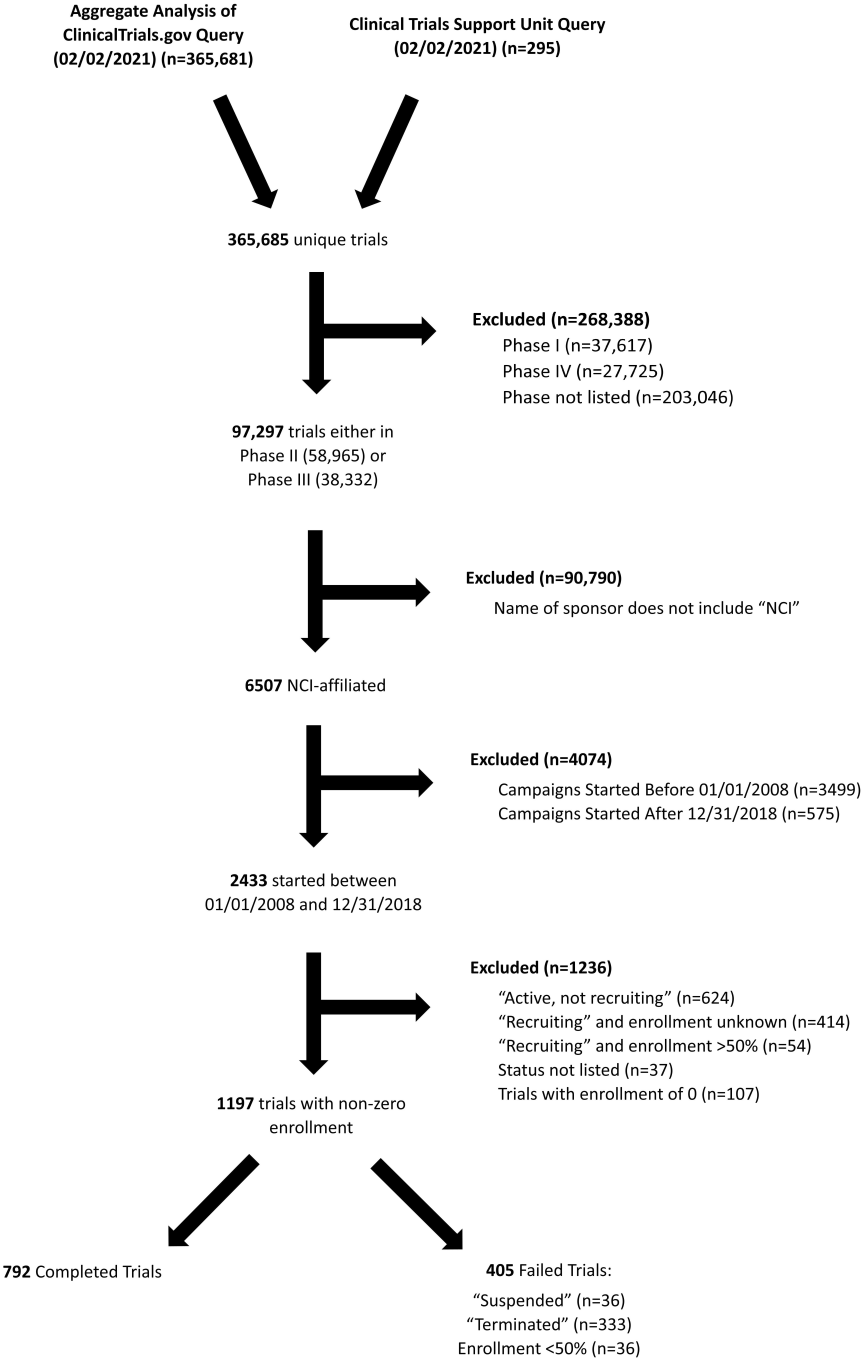
Data Selection and organization flowchart

**FIGURE 2:**
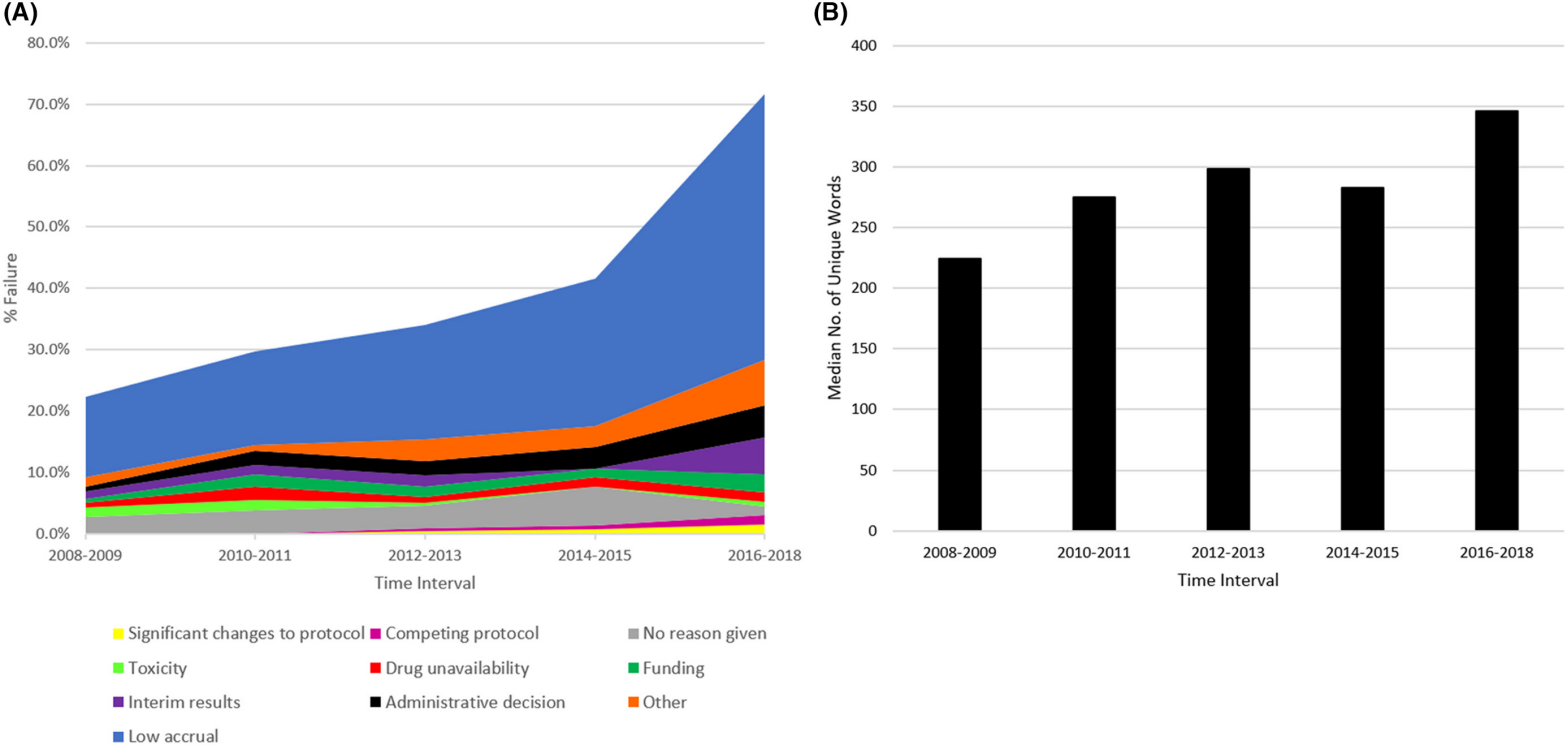
Trial failure rates and increasing eligibility criteria content among NCI‐affiliated cancer trials between January 1, 2008 and December 31, 2018 (*n* = 1197 trials). (A) Trial failure rates and reasons for failure among trials by initiation year. (B) Increase in median number of unique content words by trial initiation year

A total of 1197 trials met our inclusion criteria for analysis (Figure 1). Of these, 405 (33.8%) trials failed. The following categories were identified as the most common reasons for failure: low accrual (*n* = 231; 57.0%), an administrative decision (6.7%), poor interim results (5.4%), funding issues (4.4%), logistical challenges (4.0%), toxicity and safety concerns (3.2%), and competing clinical trials (1.0%) (Supplementary Table S1). The proportion of trials that failed due to poor accrual increased over time, while the proportion of trial failures due to non‐accrual reasons stayed fairly constant (Figure 2A). There were 42 (10.4%) trials for which no reason was given for closure and 28 (6.9%) closed for other reasons.

### 3.2 Trends in eligibility criteria content

**FIGURE 3:**
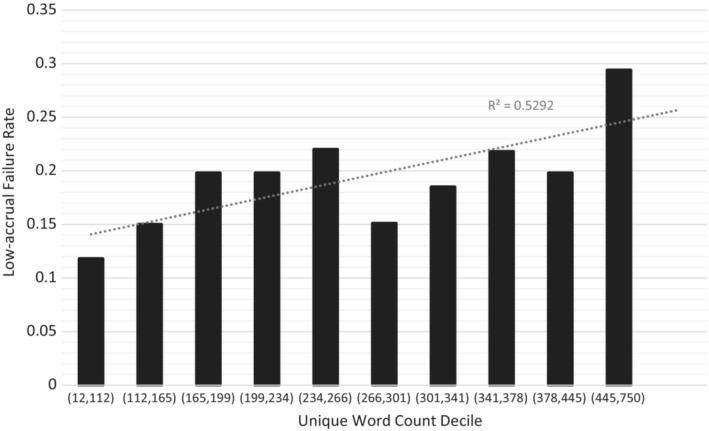
Rates of low‐accrual failure for each decile of unique word count of eligibility criteria of NCI‐affiliated cancer trials (*n* = 1197 trials)

The median number of unique content words in trial eligibility criteria increased by 95% over a 10‐year period, from 214 (IQR: 23, 289) in 2008 to 416 (IQR: 289, 514) in 2018 (*p* = 0.0008, *r* ^2^ = 0.732; Figure 2B). Trial failure rate was associated with increased unique word count decile from 11.8% in the first decile to 29.4% in the tenth decile (*r* ^2^ = 0.529, Figure 3).

### 3.3 Predictors of trial accrual failure

On multivariable analysis, unique word count decile was associated with accrual failure (OR 1.09, 95% CI: [1.03–1.15]; *p = 0*.004), as was phase (Phase III vs. Phase II, OR: 1.74, 95% CI: [1.13–2.64]; *p = 0*.01) (Table 1). Total number of eligibility criteria was also independently associated with accrual failure, though the association was not as strong as that of unique word count (Supplementary Tables S4 and S5).

**TABLE 1:** Multivariate logistic regression for accrual failure in NCI‐affiliated cancer trials between 2008 and 2018 (*n* = 1197 trials)

Variable	OR (95% CI)	*p* value
Number of unique content words (per decile)	1.09 (1.03–1.15)	0.004
Common tumors	1.13 (0.84–1.52)	0.41
Target therapy as intervention	1.32 (0.91–1.95)	0.15
Metastatic setting	1.26 (0.91–1.74)	0.16
Tissue sample required	1.00 (0.73–1.36)	0.99
Radiation therapy as intervention	0.85 (0.24–2.35)	0.78
Phase III (vs. Phase II)	1.74 (1.13–2.64)	0.01

### 3.4 Clinical categories within exclusion criteria and association with accrual failure

After removal of trials that did not have distinct exclusion criteria section in the AACT, 887 clinical trials remained for analysis. After extraction of biomedical entities and statistical analysis, we found 276 terms out of 12,870 terms that appeared more frequently in trials with accrual failures (Bonferroni‐corrected threshold of *p* < 6.72 × 10^−6^, Appendix 1). Of these, 128 terms were grouped into the following 18 categories: “age,” “allergy and immunology,” “bleeding and coagulopathy,” “cardiovascular,” “chemotherapy,” “gastrointestinal,” “reproductive,” “hematology/oncology,” “hepatic,” “imaging,” “infection,” “diabetes,” “neuropsychiatric,” “orthopedic,” “pulmonary,” “radiation therapy,” “renal,” and “surgery” (Table 2). The remaining 148 terms were excluded from categorization as they were ambiguous, nonspecific, or were effectively stop words (e.g., “course,” “criteria,” “associated with”) (Appendix 2). Following optimal fit with gradient‐boosted trees classifier, feature importance analysis demonstrated that quantity of medical terms present was the most important feature. Other important exclusion criteria categories were renal, pulmonary, immunologic, diabetic, and age restrictions (Supplementary Figure S2). Sensitivity analyses of exclusion and inclusion criteria quantified separately demonstrated a strong association between accrual failure and increasing unique content words of exclusion criteria, while association with inclusion criteria was not statistically significant (Appendix 3). Sensitivity analysis including trials with unknown phase demonstrated that a strong association between eligibility criteria unique content words and accrual failure was maintained (Appendix 4).

**TABLE 2:** Exclusion criteria biomedical terms associated with accrual failure, organized by clinical category among NCI‐affiliated trials (*n* = 887, Bonferroni‐corrected *p* value threshold for significance = 6.7186 × 10^−6^)

Category	Medical terms	Successful no. (%)	Failed no. (%)	*p* value
Reproductive	Breast, breastfeeding, cervical, childbearing, childbearing potential, contraception, female, females, lactating, mother, potential, pregnancy test, pregnant, pregnant women, women	617 (86.1%)	159 (93.5%)	9.69 E‐61
Infectious	Abscess, active, active infection, antibiotics, antiretroviral, antiretroviral therapy, hiv, human, human immunodeficiency, human immunodeficiency virus, immunodeficiency, immunodeficiency virus, infection, infections, viral, virus	585 (81.6%)	150 (88.2%)	6.17 E‐58
Hematology/oncology	Cancer, carcinoma, in situ, invasive, leukemia, malignancy, metastases, metastatic, prostate cancer, skin cancer, squamous, squamous cell, tumor	541 (75.5%)	140 (82.4%)	2.75 E‐53
Allergy and immunology	Allergic, allergic reactions, allergy, corticosteroids, hypersensitivity, immunotherapy, inflammatory, steroids	504 (70.3%)	127 (74.7%)	6.50 E‐51
Cardiovascular	Angina, angina pectoris, arrhythmia, blood pressure, cardiac, cardiac arrhythmia, cardiac disease, congestive, congestive heart failure, coronary, ejection, ejection fraction, failure, fraction, heart, heart failure, infarction, myocardial, myocardial infarction, nyha, pectoris, systolic blood pressure, unstable, unstable angina, unstable angina pectoris, venous, ventricular, ventricular arrhythmias, york, york heart	507 (70.7%)	134 (78.8%)	3.98 E‐49
Neuropsychiatric	Brain, central, central nervous system, cns, neurologic, neuropathy, nervous system, psychiatric, psychiatric illness	479 (66.8%)	123 (72.4%)	1.06 E‐47
Age	Age	488 (68.1%)	131 (77.1%)	1.08 E‐46
Surgery	Allogeneic, major surgery, resection, surgery, surgical, transplant	366 (51.0%)	94 (55.3%)	7.43 E‐37
Chemotherapy	Adjuvant, chemotherapy, mitomycin, nitrosureas	343 (47.8%)	84 (49.4%)	4.87 E‐36
Radiation therapy	Radiation, radiation therapy, radiotherapy	344 (48.0%)	95 (55.9%)	1.43 E‐32
Hepatic	Bilirubin, hepatic, hepatitis, liver	312 (43.5%)	84 (49.4%)	2.16 E‐30
Gastrointestinal	Bowel, gastrointestinal, obstruction, oral, perforation, swallow, ulcer	306 (42.7%)	86 (50.6%)	1.10 E‐28
Bleeding and coagulopathy	Anticoagulation, bleeding diathesis, wound	129 (18.0%)	36 (21.2%)	4.49 E‐13
Renal	Creatinine, renal	133 (19.4%)	47 (27.6%)	1.52 E‐11
Pulmonary	Pulmonary	179 (18.5%)	44 (25.9%)	2.24 E‐11
Orthopedic	Bone, bone fracture, fracture	119 (16.6%)	37 (21.8%)	5.19 E‐11
Imaging	Imaging, mri	89 (12.4%)	22 (12.9%)	2.03 E‐10
Diabetes	Diabetes	107 (14.9%)	35 (20.6%)	1.52 E‐09
Median no. of terms	All terms (*n* = 128)	93	99	2.39 E‐03

## 4 DISCUSSION

In this large study of NCI‐affiliated cancer trials, we found that the incidence of accrual failures is rising and strongly associated with increasing eligibility criteria content. This association of content growth with accrual failure persisted after adjustment for multiple known contributors to accrual failure. We additionally demonstrated how natural language processing and machine learning can be leveraged to determine specific exclusionary biomedical terms that are most associated with accrual failure. We found that, more than the quality of the criteria, it was the *quantity* of eligibility criteria content that most strongly associated with accrual failure. We interrogated several surrogates for criteria complexity and robustly demonstrated their association with accrual failure: unique content word decile, number of criteria, and the number of medical term categories represented in the exclusion criteria. Our study advances the understanding of eligibility criteria as barriers to trial accrual and supports ongoing efforts to simplify eligibility criteria.

Our findings of increased accrual failures are consistent with prior studies. In 2014, Stenslund et al. reported that poor enrollment was the most common cause for failure among the clinical cancer trials initiated by the NCI Cooperative Group between 2005 and 2011, responsible for 36.3% of these 935 failures.^18^

The causes of increased accrual failures are likely multifactorial and may include the increasingly complex and precision‐based nature of oncology care, the outpacing of trials compared to number of patients, the funding‐related issues for non‐industry sponsored trial, and eligibility criteria characteristics.^19^, ^20^, ^21^ Recently, there has been an increasing number of precision oncology trials, which inherently come with more stringent eligibility requirements. Our study suggests that eligibility content quantity, in and of itself, as a surrogate for eligibility exclusivity, is associated with poor accrual. Given that each criterion introduces a potential restriction on enrollment population that trial staff members must assess for each patient prior to registration, this association with poor accrual is plausible. Intriguingly, the data suggest no clear threshold for risk of accrual failure with unique content words, but a relatively linear correlation. This suggests that most, if not all, cancer trials may benefit from streamlining of eligibility criteria to improve trial accrual, particularly in when it comes to baseline patient‐centric (as opposed to tumor‐centric) factors, such as comorbidities and demographics.

Organizing content into medical entity categories allowed us to probe the impact of specific exclusion criteria themes on accrual. NLP allowed for the identification of lexical associations with low‐accrual failure—associations that corroborate contemporary research and expert opinion. Exclusions pertaining to age, HIV status, and renal function, for example, are all identified as traditional areas of exclusion in the 2017 ASCO‐*Friends* recommendations for enrollment expansion, and each individually appeared more frequently among the exclusion criteria of low‐accruing trials in our study.^22^, ^23^, ^24^ Likewise, reproductive factors, like pregnancy and breastfeeding, were more represented in the exclusion criteria of low‐accruing trials. Demographic disparities, including by race and geographic region, between the participants in clinical cancer trials and the rest of the cancer population are well‐documented.^25^, ^26^ In 2004, Murthy et al. found lower enrollment rates among elderly patients and females compared to their younger and male counterparts.^27^ More recently, Ludmir et al. found cancer trial participants to be 6.49 years younger on average than the general cancer population and the disparity to be growing by about 10 weeks each year.^21^ We additionally identified accrual failure associations with several restriction categories that had not been documented previously, such as diabetes and neuropsychiatric conditions. These themes and the others identified in our analysis should be explored in greater depth in future work.

Our findings not only largely support ongoing efforts by national organizations to reduce barriers to cancer trial enrollment, but also highlight that more may be needed to reverse the alarming trend of increasing accrual failure. Beyond the general simplification of criteria, our results encourage trialists to consider certain clinical and demographic characteristics of the population, such as age, sex, and comorbidity restrictions, when designing eligibility criteria. Our results demonstrated that the presence of renal, pulmonary, immunologic, and diabetic restrictions was particularly associated with accrual failure and highlight that there should be attempts to include patients with these comorbidities if sufficiently safe to do so. When lab cut‐offs are placed in the exclusion criteria, levels that balance inclusivity and safety are recommended.

This study is not without limitations. While we found a robust association between eligibility criteria growth and accrual failure, we were not practically able to control for all potential confounders. Given the retrospective nature of the study, we cannot prove a causal relationship between eligibility criteria and accrual failure, and as discussed above, there are many contributing factors to accrual failure. Despite this, there is a plausible basis for the link between eligibility criteria complexity and accrual, and we have mitigated the potential for confounding by limiting our sample to Phases II and III, interventional NCI‐affiliated trials and conducting a multivariable analysis that controlled for numerous established risk factors of low accrual. While reducing confounding, we recognize that this limited sample makes the findings less generalizable to other type of cancer trials, including single‐institution and industry‐sponsored trial, as well as those enrolling pediatric populations or rare cancers, and this should be explored in future studies. Additionally, it is not possible to track accrual in real time via the AACT data. Thus, while our definition of accrual failure (<50% 2‐year post‐initiation) is a definition based on best available evidence, it may have overestimated accrual failure for trials with initially slow, but eventually successful enrollment, particularly for trials initiated in the later years of the study period. Notwithstanding, low enrollment was the most common reason for trial failure every year between 2008 and 2018, and while other causes of failure remained relatively stable, low‐accrual failure showed a positive trend between 2008 and 2016. This remained the case during the sensitivity analysis, using a more liberal definition of “completion” (Appendix 2). Regarding the analysis of medical term categories, the NLP tools we used are limited in their ability to capture contextual relationships between words and sentences that may modify the effects of specific terms and categories on accrual. More advanced applications of NLP, such as use of deep learning‐based language models pretrained on large textual datasets may provide a means to incorporate more complex text relationships.^28^ Despite the limitations, we believe these findings highlight the strong association of eligibility criteria on patient accrual and provide valuable information for patients, investigators, government agencies, and advocates involved in the design and execution of clinical trials.

## 5 CONCLUSIONS

Eligibility criteria content among NCI‐affiliated clinical cancer trials is increasing in quantity and complexity, and this growth is strongly associated with trial accrual failure. Certain clinical exclusions, such as organ dysfunction, age, and neuropsychiatric conditions, were identified as particularly associated with accrual failure. These findings support national recommendations to simplify eligibility criteria. They suggest that further efforts may be needed to actualize these recommendations and improve cancer trial accrual.

## AUTHOR CONTRIBUTIONS

Writing original draft—JSP. Writing, editing, and revision—JSP, BHK, DP, DSB, HJWLA, SBJ. Methodology—BHK, JSP. Data collection—JSP, BHK. Data analysis—JSP, BHK.

## FUNDING INFORMATION

Not applicable.

## ETHICS STATEMENT

This study did not involve interaction with human subjects and was therefore deemed IRB exempt.

## Supporting information


Appendix S1

Appendix S2

Appendix S3

Appendix S4

## Data Availability

All data are publicly accessible via the AACT and CTSU. Derived datasets used in the study will be shared on reasonable request to the corresponding author.
