# Supplementary material for: Growth in eligibility criteria content and failure to accrue among National Cancer Institute (NCI)‐affiliated clinical trials
Source: Cancer Med. 2022 Nov 18;12(4):4715–24. doi: 10.1002/cam4.5276 (PMC9972031; doi:10.1002/cam4.5276)
Supplement: Supplementary file 1 — Appendix S1 [file CAM4-12-4715-s004.docx]

**Appendix 1**

The Aggregate Analysis of ClinicalTrials.gov Clinical Trials Transformation Initiative (AACT-CTTI) makes available all information about every study registered on clinicaltrials.gov^1^. The database is updated daily and downloadable in discrete folders that contain specific protocol and trial information which can be processed and merged.

The AACT files with their corresponding informational variables used in this study accompanied by explanatory definitions drawn from the AACT Comprehensive Data Dictionary:

1. brief_summaries:
   1. description:
      1. A brief description of the trial (5000 characters or less), including its hypothesis, written in plain language.
2. browse_conditions
   1. downcase_mesh_terms:
      1. Terms from the Medical Subject Heading (MeSH) thesaurus describing the clinical trial conditions (e.g. “cancer”, “lymphoma”).
3. browse_interventions
   1. downcase_mesh_terms:
      1. Terms from the MeSH thesaurus describing the intervention used in the trial.
4. drop_withdrawals
   1. count:
      1. The number of subjects withdrawn from the clinical trial.
   2. reason:
      1. The reason for each subject’s withdrawal from the trial.
5. eligibilities
   1. criteria:
      1. A summary of the clinical trial’s eligibility criteria in 15,000 characters or less. Formatting the criteria as lists of inclusion and exclusion criteria is recommended but not required.
6. interventions
   1. description:
      1. A detailed description of the interventions used in each arm of the clinical trial in 999 characters or less.
   2. intervention_type:
      1. The intervention studied; either drug, device, biological/vaccine, procedure/surgery, radiation, behavioral, genetic, dietary supplement, or other
   3. name:
      1. The name of the intervention.
7. sponsors
   1. agency_class:
      1. The general category of the study sponsor (e.g., “Industry”, “U.S. Fed”, “NIH”).
   2. lead_or_collaborator:
      1. Defines whether the named sponsor is playing a collaborating or leadership role.
   3. name:
      1. The name of the leader or collaborator
8. studies
   1. completion_date:
      1. The date all data collection ends
   2. nct_id:
      1. A unique identifying code given assigned to each trial registered at clinicaltrials.gov; “NCT-” followed by eight digits
   3. official title:
      1. The official title of the study in 600 characters or less.
   4. official_status:
      1. The recruitment status of the study. Either “Not yet recruiting”, “Recruiting”, “Enrolling by invitation”, “Active, not recruiting”, “Completed”, “Suspended”, “Terminated”, “Withdrawn”. See “Overall Status Definitions” below for more information*.
   5. phase:
      1. Clinical trial phase
   6. start_date:
      1. The date a clinical trial began in “month/day/year” format. If only the month and year are provided then the last day of the month is used.
   7. type:
      1. The type of clinical trial; either “Interventional” or “Observational”.
   8. why_stopped:
      1. A brief statement (160 characters or less) explaining why a clinical trial was suspended, withdrawn, or terminated

***Overall Status Definitions:**

The AACT Comprehensive Data Dictionary defines each recruitment status as follows:

- Not yet recruiting: study subjects are not yet being recruited
- Recruiting: subjects are being recruited
- Enrolling by invitation: subjects are or will be chosen from a predetermined population
- Active, not recruiting: the trial is ongoing but no subjects are currently being recruited
- Completed: the trial completed normally and no subjects are being recruited or treated
- Suspended: recruitment or enrollment of research subjects stopped early but may resume
- Terminated: recruitment or enrollment of research subjects halted prematurely and will not resume; no subjects are being followed or treated
- Withdrawn: the trial stopped before enrolling its first research subject

**Search Terms:**

Supplementary Tables 1 and 2 layout the search terms used to classify clinical trials by their reasons for failure, by searching in “why_stopped”, and the primary cancer type they studied, by searching the “downcase_mesh_terms” in “browse_conditions”.

**Supplementary Table 1.**

| Reason for Failure | Search Terms | No. of Clinical Trials |
| --- | --- | --- |
| Low accrual | "not enough patients", "lack of accrual", "lack of enrollment", "lack of patient", "lack of inclusion", "lack of recruitment", "lack of patients", "lack of referrals", "lack of study population", "poor", "slow", "stopped", "accru", "acru", "enrol", "recruit", "rate", "longer", "unable", "time", "low", "zero", "expectations", "challenges", "participants", "issue", "patients", "insufficient enrollment", "insufficient accrual", "insufficient recruitment", "subjects" | 231 |
| Administrative decisions | "administrative", "business", "company", "corporate", "sponsor", "commercial", "sponsor's", "funder", “cancelled”, “committee”, “principal investigator”, “pi” | 26 |
| Poor interim results | "interim", "lack of efficacy", "discouraging results", "analysis", "assessment" | 18 |
| Funding issues | “funding”, “funds'', “resources” | 18 |
| Drug Availability and Logistics | "logistics", "drug supply", "drugs unavailable", "drug", "fda", "study drug", "not available", "study agent", "no longer available" | 17 |
| Safety and toxicity concerns | "toxic", "toxicity", "toxicities", "deaths", "fatal", "patient~ safety", "died", "safety issue", "safety monitoring", "safety data", "safety measures", "safety related", "safety reasons", "safety concern", "safety and efficacy" | 9 |
| Competition with Another Study | “competing”, “new study to open soon”, “a more highly selected protocol” | 4 |
| Significant Study Changes | “revision”, “modification”, “amendment” | 4 |

**Supplementary Table 2.**

| Cancer Type | Search Terms | No. of Trials |
| --- | --- | --- |
| Anal | “anal”, “anus” | 3 |
| Bladder and Urethral | “bladder”, “urothe”, “ureth”, “urinary” | 9 |
| Biliary | “bile”, “biliary” | 0 |
| Brain and CNS | “brain”, “central nervous system”, “glioblastoma”, “glioma”, “cns” | 41 |
| Breast | “breast” | 90 |
| Cervical and Fallopian tube | “cervical”, “cervix”, “fallopian” | 10 |
| Colorectal | “colon”, “colorectal”, “rectal”, “rectum” | 31 |
| Endometrial | “endometrial”, “uterine”, “uterus” | 7 |
| Esophageal | “esophageal” | 4 |
| Kidney | “kidney”, “renal” | 9 |
| Larynx | “larynx”, “laryngeal” | 0 |
| Leukemia | “leukemia”, “hemato” | 96 |
| Liver | “liver”, “hepatic”, “hepato” | 6 |
| Lung and Broncheal | “lung”, “bronch” | 78 |
| Non-hodgkins Lymphoma | “non-hodgkin”, “nonhodgkin”, “mantle cell”, “t-cell lymphoma”, t cell lymphoma”, “b-cell lymphoma”, “b cell lymphoma”, “burkitt”, “lymphoctic lymphoma” | 8 |
| Non-specific | “unknown primary” | 0 |
| Melanoma | “melanoma” | 72 |
| Myeloma | “myeloma”, “plasmacytoma” | 51 |
| Oral | “oral”, “mouth”, “pharynx”, “salivary gland” | 3 |
| Ovarian | “ovar” | 12 |
| Pancreatic | “pancrea” | 20 |
| Prostate | “prostate”, “prostatic” | 74 |
| Gastric | “gastric”, “stomach | 3 |
| Testicular | “testi” | 5 |
| Vulvar | “vulva” | 0 |
| Lymphoma | “lymphoma”, “hemato” | 96 |
| Sarcoma | “sarcoma” | 38 |
| Neuroblastoma | “neuroblastoma” | 4 |
| Mesothelioma | “mesothelioma” | 9 |
| Head and Neck | “head”, “neck”, “h&n” | 9 |
| Thymoma | “thymoma”, “thymic” | 1 |
| Penile | “penile” | 0 |
| Merkel cell | “merkel” | 3 |
| Trophoblastic | “trophoblast” | 0 |
| Neuroendocrine | “neuroendocrine”, “islet cell”, “net” | 4 |
| Gastrointestinal Stromal Tumor | “gastrointestinal”, “stromal”, “gist” | 5 |
| Waldenstrom | “waldenstrom”, “macroglobinemia” | 1 |

| Reasons for Annual Failure | Failure Rate No. (%)  (n= 405) |
| --- | --- |
| Low Accrual | 231 (57.0%) |
| No reason given | 42 (10.4%) |
| Administrative Decision | 27 (6.7%) |
| Poor Interim results | 22 (5.4%) |
| Funding Issues | 18 (4.4%) |
| Drug Unavailability and Logistics | 16 (4.0%) |
| Safety and Toxicity Concerns | 13 (3.2%) |
| Competition with Another Study | 4 (1.0%) |
| Significant Study Changes | 4 (1.0%) |
| Other* | 28 (6.9%) |

**Supplemental Table 3.** Reasons for the Failure Rates Among NCI-Affiliated Cancer Trials between 1 January 2008 and 31 December 2018

*Includes reasons that did not reach a 1% rate threshold, including “closed prematurely”, “halted prematurely and will not resume”, and “research cancelled”.

**Supplementary Figure 1.** Median Number of Bullets in Eligibility Criteria of NCI-affiliated Cancer Trials Each Year (*r^2^=.*239, *p*=.127)

**Results for Univariate Logistic Regression for Accrual Failure and Median Number of Bullet Points:**

- OR (95% CI): 1.010 (1.002 – 1.017)
- P value: 0.012

**Supplementary Table 4.**

| Multivariate Logistic Regression for Accrual Failure | OR (95% CI) | P value |
| --- | --- | --- |
| Number of Bullet Points | 1.01 (1.00 – 1.02) | .03 |
| Common tumors | 1.09 (0.81 – 1.46) | .56 |
| NCI-approved targeted treatment as intervention | 1.32 (0.91 – 1.94) | .15 |
| Metastatic disease | 1.30 (0.94 – 1.79) | .11 |
| Tissue sample required | 1.04 (0.76 – 1.41) | .83 |
| Radiation therapy as intervention | 0.88 (0.25 – 2.41) | .82 |
| Phase III | 1.60 (1.05 – 2.41) | .03 |

**Supplementary Table 5.** Characteristics of NCI-affiliated Clinical Cancer Trials That Failed Due to Low Accrual and Those That Completed Successfully Between 2008 and 2018

| Variable | Completed Trials (n=792) | Accrual Failures (n=231) | P value |
| --- | --- | --- | --- |
| No. of unique content words (Median [Interquartile Range]) | 708 (456 – 1078.5) | 812 (538 – 1230) | .001 |
| Common cancers | 333 (42.0%) | 102 (44.2%) | .57 |
| NCI-approved targeted treatment as intervention | 611 (77.1%) | 191 (82.7%) | .09 |
| Metastatic disease | 188 (23.7%) | 70 (30.3%) | .05 |
| Tissue sample required | 310 (39.1%) | 100 (43.3%) | .29 |
| Radiation therapy as intervention | 15 (2.0%) | 4 (1.7%) | 1.0 |
| Phase III | 101 (12.8%) | 36 (15.6%) | .27 |

**Analysis of Medical Terms:**

We utilized the ScispaCy “en_core_sci_md” pipeline model which contains 50,000 biomedical word vectors. The exclusion criteria were tokenized. In this step, en_core_sci_md was calibrated to identify medical terms consisting of five words or less within each clinical trial’s exclusion criteria text. 12,870 unique n-grams were identified. These exclusion criteria n-grams were then formatted into a document-term matrix. To improve processing speed, columns representing n-grams that only appeared once in the entire matrix were deleted reducing the number of n-grams in the analysis to 5905. This was justified since n-grams this infrequent did not have a distribution that differed significantly between the low-accruing trials and successful trials. The resulting document-term matrix was then split into two—one containing clinical trials that failed due to low accrual and the other containing successful trials.

To determine which medical terms appeared significantly more often in low-accruing clinical trials, the values in the two term-document matrices were binarized; values greater than zero were converted to “1” and values of zero were left as “0”. The total number of exclusion criteria in which each term appeared was then summed for the successful and low-accruing matrices, respectively.

A two-sided χ^2^ test of independence was then performed for each term with post-hoc Bonferroni correction for multiple hypothesis testing. Medical terms that appeared more frequently in accrual failure trials were then grouped into thematic categories by consensus of two study authors (JSP, BHK). A two-sided χ^2^ test of independence with Bonferroni correction (n=19) was then performed to evaluate differences within each category between accrual failures and successful trials.

**List of Terms:**

The following 276 terms were significantly more represented in the text of exclusion criteria of clinical trials that failed due to low accrual. Words with significantly different representation in the two groups are highlighted in **red**:

1. abnormalities
2. **abscess**
3. **active**
4. **active infection**
5. acute
6. **adjuvant**
7. administered
8. administration
9. adverse
10. adverse events
11. **age**
12. agent
13. agents
14. **allergic**
15. **allergic reactions**
16. **allergy**
17. **allogeneic**
18. **angina**
19. **angina pectoris**
20. **antibiotics**
21. **anticoagulation**
22. **antiretroviral**
23. **antiretroviral therapy**
24. **arrhythmia**
25. associated with
26. **bilirubin**
27. biologic
28. biologic composition
29. biopsy
30. **bleeding diathesis**
31. **blood**
32. **blood pressure**
33. **bone**
34. **bone fracture**
35. **bowel**
36. **brain**
37. **breast**
38. **breastfeeding**
39. **cancer**
40. **carcinoma**
41. **cardiac**
42. **cardiac arrhythmia**
43. **cardiac disease**
44. cell
45. **central**
46. **central nervous system**
47. **cervical**
48. **chemotherapy**
49. **childbearing**
50. **childbearing potential**
51. chronic
52. clinical
53. clinical trial
54. clinically
55. clinically significant
56. **cns**
57. combination
58. completion
59. compliance
60. composition
61. compounds
62. concomitant
63. concurrent
64. condition
65. conditions
66. **congestive**
67. **congestive heart failure**
68. consent
69. **contraception**
70. controlled
71. **coronary**
72. **corticosteroids**
73. course
74. **creatinine**
75. criteria
76. ctcae
77. current
78. cyp3a4
79. days
80. **diabetes**
81. diagnosed
82. diagnosis
83. discontinued
84. disease
85. disease free
86. disorders
87. documented
88. dose
89. doses
90. drug
91. drugs
92. duration
93. dysfunction
94. effects
95. **ejection**
96. **ejection fraction**
97. eligible
98. entry
99. evaluation
100. event
101. events
102. evidence
103. excluded
104. exclusion
105. **failure**
106. **female**
107. **females**
108. **fraction**
109. **fracture**
110. function
111. **gastrointestinal**
112. grade
113. healing
114. **heart**
115. **heart failure**
116. **hepatic**
117. **hepatitis**
118. history
119. **hiv**
120. **human**
121. **human immunodeficiency**
122. **human immunodeficiency virus**
123. **hypersensitivity**
124. illness
125. **imaging**
126. **immunodeficiency**
127. **immunodeficiency virus**
128. **in situ**
129. increased
130. inducers
131. **infarction**
132. **infection**
133. **infections**
134. **inflammatory**
135. informed
136. inhibitor
137. inhibitors
138. injury
139. interactions
140. intercurrent
141. intercurrent illness
142. interfere with
143. interval
144. **invasive**
145. investigational
146. investigational agent
147. investigator
148. **lactating**
149. **leukemia**
150. limit
151. **liver**
152. low
153. major
154. **major surgery**
155. **malignancy**
156. marrow
157. medical
158. medication
159. medications
160. men
161. **metastases**
162. **metastatic**
163. method
164. **mitomycin**
165. months
166. **mother**
167. **mri**
168. **myocardial**
169. **myocardial infarction**
170. negative
171. **nervous system**
172. **neurologic**
173. **neuropathy**
174. **nitrosoureas**
175. non
176. nursing
177. **nyha**
178. **obstruction**
179. ongoing
180. opinion
181. **oral**
182. organ
183. patient
184. patients
185. **pectoris**
186. **perforation**
187. period
188. physician
189. poor
190. positive
191. **potential**
192. **pregnancy test**
193. **pregnant**
194. **pregnant women**
195. presence
196. previous
197. primary
198. prior
199. procedure
200. procedures
201. progression
202. progressive
203. **prostate cancer**
204. protocol
205. **psychiatric**
206. **psychiatric illness**
207. **pulmonary**
208. **radiation**
209. **radiation therapy**
210. **radiotherapy**
211. randomization
212. reactions
213. recovered
214. regimen
215. registration
216. **renal**
217. requirements
218. **resection**
219. risk
220. safety
221. screening
222. serious
223. serum
224. severe
225. significant
226. situations
227. skin
228. **skin cancer**
229. small
230. social
231. social situations
232. **squamous**
233. **squamous cell**
234. stable
235. standard
236. status
237. **steroids**
238. study
239. study requirements
240. study treatment
241. **surgery**
242. **surgical**
243. **swallow**
244. symptomatic
245. symptoms
246. syndrome
247. systemic
248. **systolic blood pressure**
249. test
250. therapeutic
251. therapy
252. time
253. **transplant**
254. treated
255. treated with
256. treating
257. treatment
258. **tumor**
259. **ulcer**
260. uncontrolled
261. **unstable**
262. **unstable angina**
263. **unstable angina pectoris**
264. untreated
265. **venous**
266. **ventricular**
267. **ventricular arrhythmias**
268. **viral**
269. **virus**
270. weeks
271. weight
272. **women**
273. **wound**
274. years
275. **york**
276. **york heart**

**Gradient-boosted Trees Classifier Method Details:**

We utilized the XGboost package in Python v3.8. After splitting off 80% of the dataset for development and validation, we conducted a grid search with 5-fold cross validation, utilization the Scikit learn package with Python script as follows:

model = XGBClassifier(objective= 'binary:logistic', seed=42, nthreads=4, use_label_encoder=False, scale_pos_weight=3)

parameters = {

'max_depth': range (2, 10, 1),

'n_estimators': range(60, 220, 40),

'learning_rate': [0.3, 0.2, 0.1],

'scale_pos_weight': [1, 3, 5, 7]

}

grid_search = GridSearchCV(

estimator=model,

param_grid=parameters,

scoring = 'roc_auc',

n_jobs = 10,

cv = 5,

verbose=False

)

grid_search.fit(X_features, y_train)

Following the grid search, the optimal hyperparameters selected were:

XGBClassifier(base_score=0.5, booster='gbtree', colsample_bylevel=1,

colsample_bynode=1, colsample_bytree=1, gamma=0, gpu_id=-1,

importance_type='gain', interaction_constraints='',

learning_rate=0.1, max_delta_step=0, max_depth=3,

min_child_weight=1, missing=nan, monotone_constraints='()',

n_estimators=60, n_jobs=8, nthreads=4, num_parallel_tree=1,

random_state=42, reg_alpha=0, reg_lambda=1, scale_pos_weight=7,

seed=42, subsample=1, tree_method='exact',

use_label_encoder=False, validate_parameters=1, verbosity=None)

Following model fitting, testing was carried out on the 20% hold-out set. The XGboost plot_importance function was used to generate a plot of the variables sorted by Gini importance.

**Supplementary Figure 2.** Feature Importance of Medical Term Categories in Exclusion Criteria in Modeling the Low-accrual Failure of NCI-Affiliated Cancer Trials

Sources:

1. AACT Database | Clinical Trials Transformation Initiative. Accessed June 2, 2021. https://aact.ctti-clinicaltrials.org/
